# Supplementary material for: Predictive prioritization of enhancers associated with pancreatic disease risk
Source: Cell Genom. 2025 Oct 16;6(1):101040. doi: 10.1016/j.xgen.2025.101040 (PMC12926207; doi:10.1016/j.xgen.2025.101040)
Supplement: Document S1. Figures S1–S11, Tables S1, S11, and S12, and consortia member lists [file mmc1.pdf]

Cell Genomics, Volume *6*

Supplemental information

Predictive prioritization of enhancers associated with pancreatic disease risk

Li Wang, Songjoon Baek, Gauri Prasad, John Wildenthal, Konnie Guo, David Sturgill, Thucnhi Truongvo, Erin Char, Gianluca Pegoraro, Katherine McKinnon, The Pancreatic Cancer Cohort Consortium, The Pancreatic Cancer Case-Control Consortium, Jason W. Hoskins, Laufey T. Amundadottir, and H. Efsun Arda

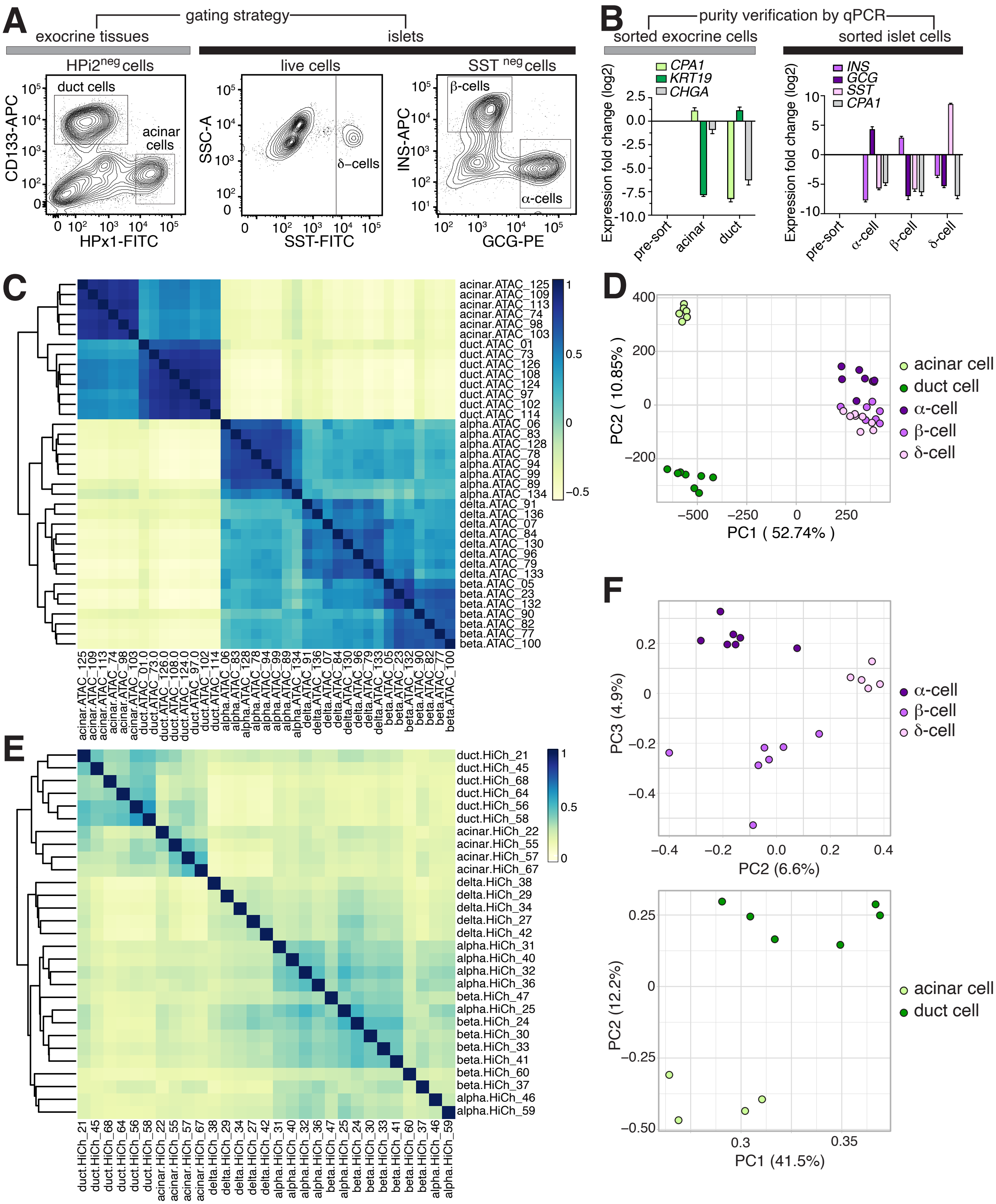

Figure S1 (related to Figure 1). Sorting strategy, data quality and donor reproducibility.
(A) FACS plots showing gating of pancreas cell populations (see Table S2). (B) Bar plots of marker gene enrichment/depletion in purified populations determined by qPCR, normalized to pre-sorted cells. Error bars, standard deviation. (C) Heat map of ATAC-seq sample clustering by Pearson correlation of accessibility profiles. (D) PCA of ATAC-seq samples, colored by cell type. (E) Heat map of HiChIP sample clustering by Pearson correlation of loop profiles. (F) PCA of HiChIP samples, colored by cell type.

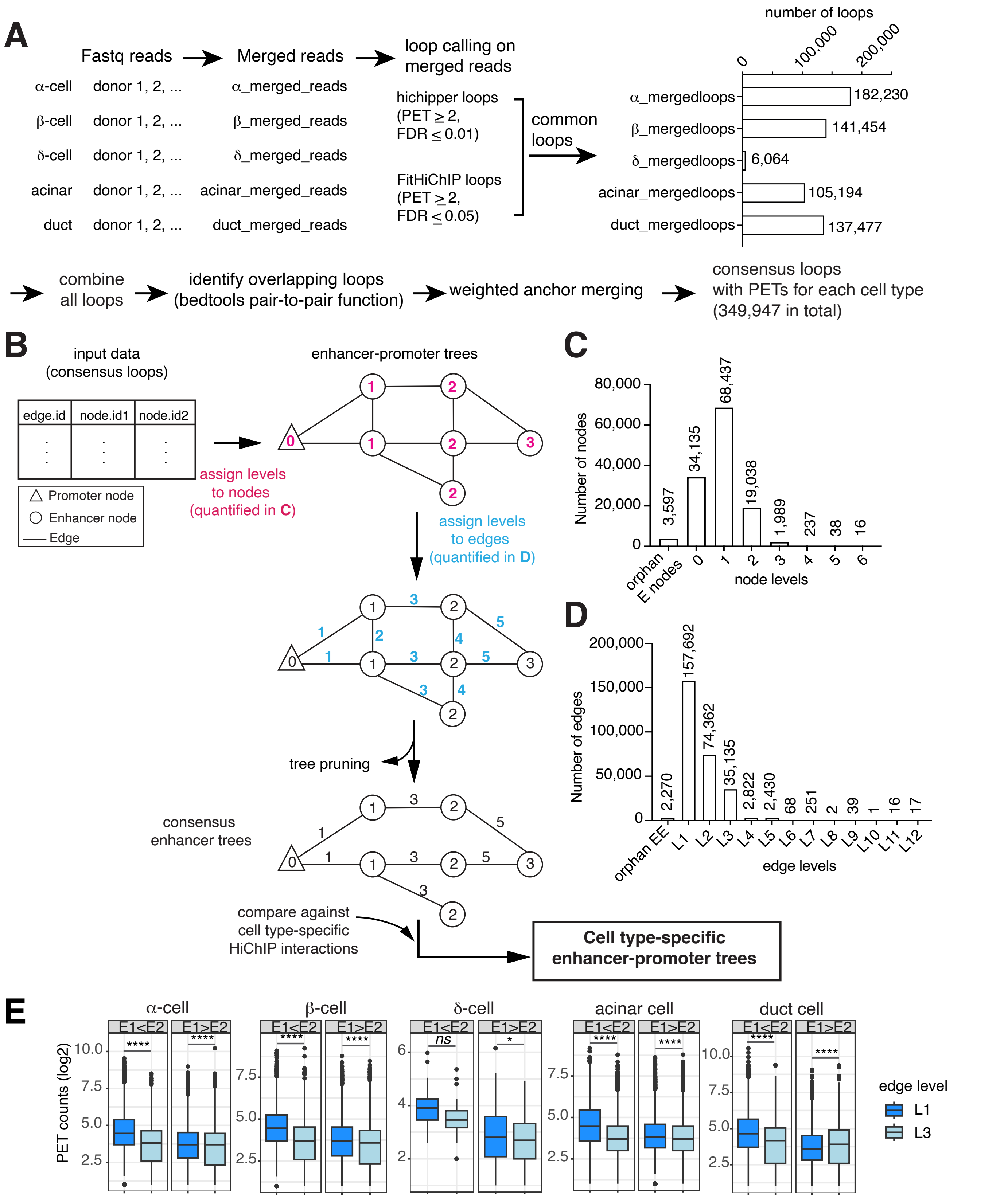

Figure S2 (related to Figure 2). Construction of enhancer trees.
(A) Flowchart of consensus loop development. (B) Schematic of enhancer tree construction from consensus loops. (C–D) Bar graphs of node (C) and edge (D) distributions by connectivity level before pruning. (E) Interaction frequency stratified by E1/E2 distance from promoters. Mann-Whitney test: **** $P < 0.0001$; * $P < 0.05$; ns, not significant. Box plots show the median line, interquartile range box, and whiskers extend to 1.5× IQR; points beyond are plotted as outliers.

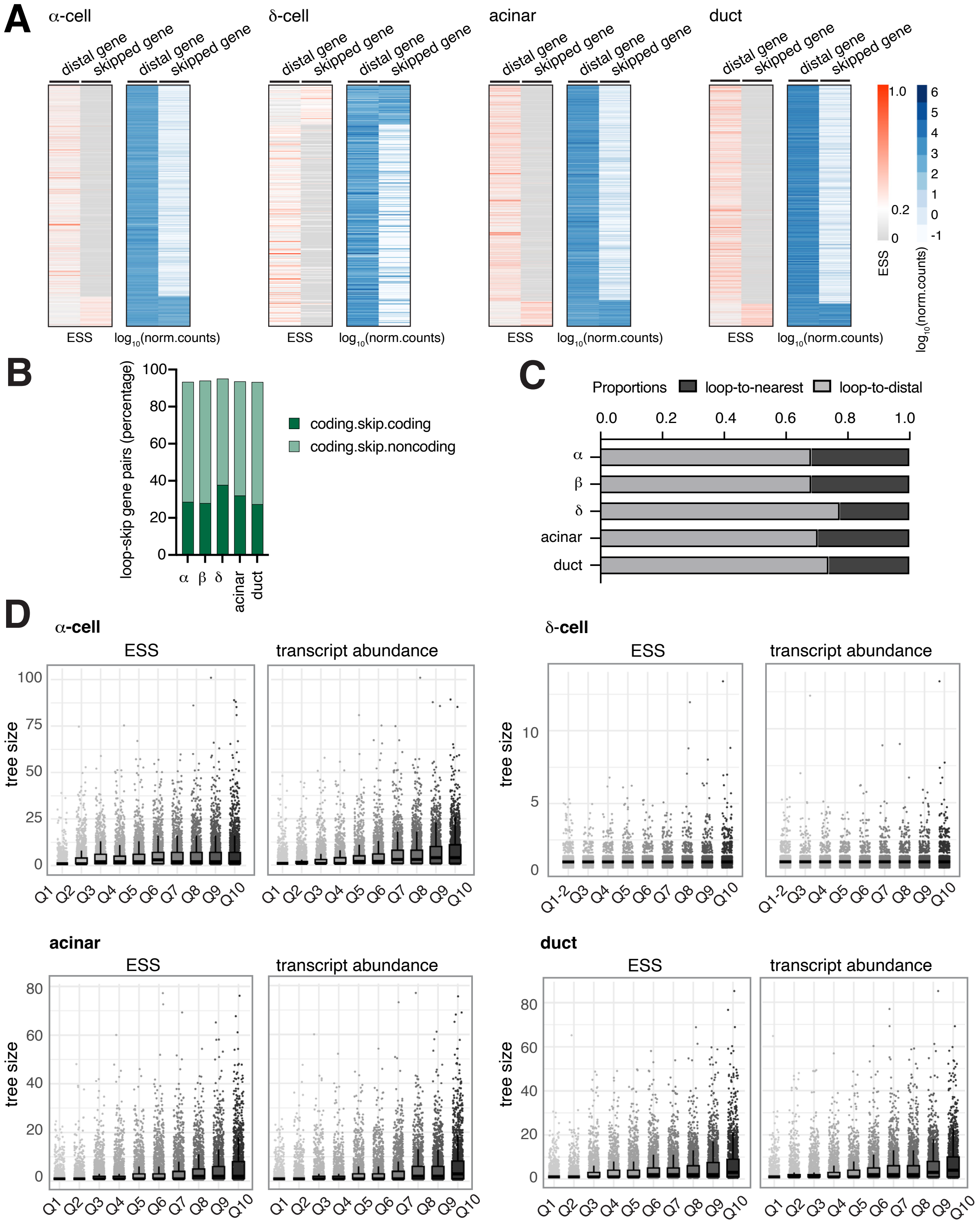

Figure S3 (related to Figure 2). Characterization of enhancer trees. (A) Heat maps of expression specificity score (ESS, red) and abundance (blue) of looped or skipped genes; each row is a gene pair looped to or skipped by the same enhancer in α-, δ-, acinar, or duct cells. (B) Distribution of gene pair types in distal looping vs skipped interactions. Dark green, both genes coding (coding.skip.coding); light green, distal gene coding, skipped gene noncoding (coding.skip.noncoding). (C) Fraction of enhancers looping to nearest (dark grey) or distal (light grey) genes, excluding noncoding genes. (D) Box plots of ESS, transcript abundance, and enhancer tree size divided across quantiles. Whiskers extend to non-outlier data, all data points are overlaid. δ-cell enhancer-promoter trees exhibit fewer interactions due to substantially lower cell yields and reduced chromatin input available for HiChIP. This technical limitation results in smaller tree sizes and less pronounced relationships between tree size, expression specificity scores, and transcript abundance compared to other cell types.

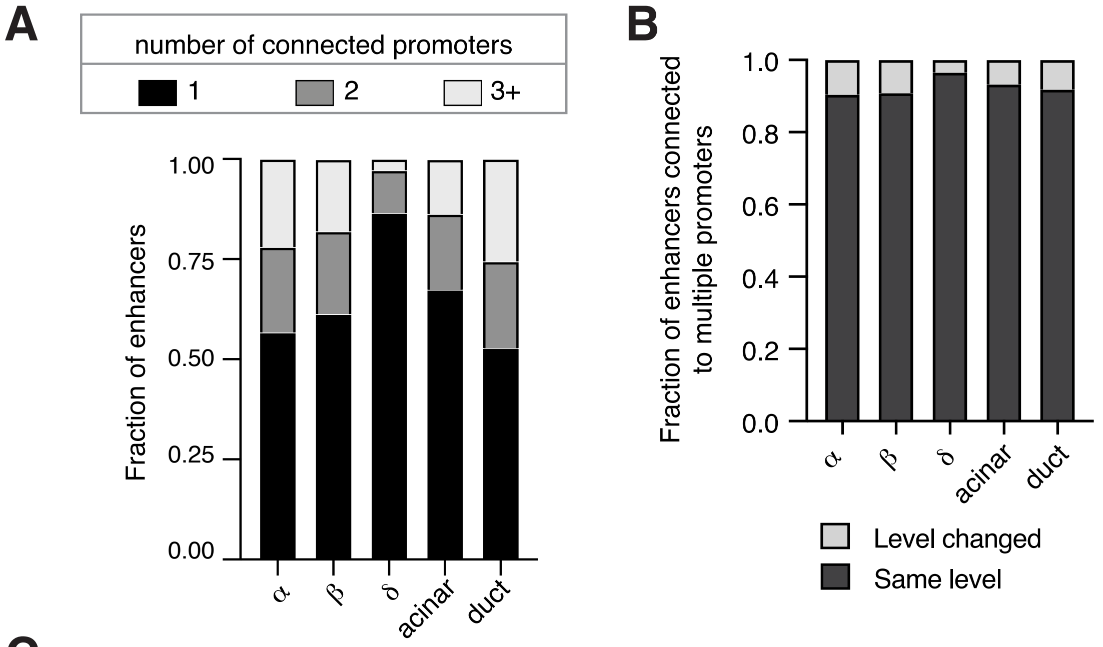

C

	α cell trees	β cell trees	acinar cell trees	duct cell trees
in forest	14813	13354	10094	12134
not in forest	744	922	855	349

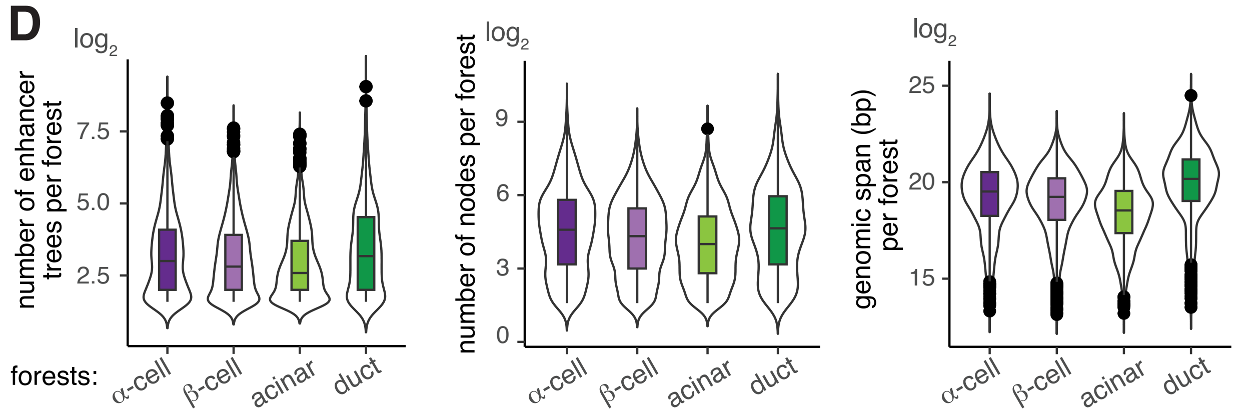

Figure S4 (related to Figure 3). Enhancer forest connectivity and topology.
(A) Bar graph shows fraction of enhancers connecting to one, two, three or more promoters quantified in each cell type. (B) Among the enhancers that connect to multiple promoters, the bar graph shows the fraction that change levels. For example, an E_1 enhancer in one tree may remain E_1 in another tree, or change to E_2, and vice versa. (C) Table summarizing the number of trees belonging to a forest versus those that are not connected, stratified by cell type. (D) Violin plots show forest level features for each cell type. From left to right: number of trees, total number of nodes and the genomic span per forest. Embedded box plots show the median line, interquartile range box, and whiskers extend to 1.5× the IQR; points beyond are plotted as outliers.

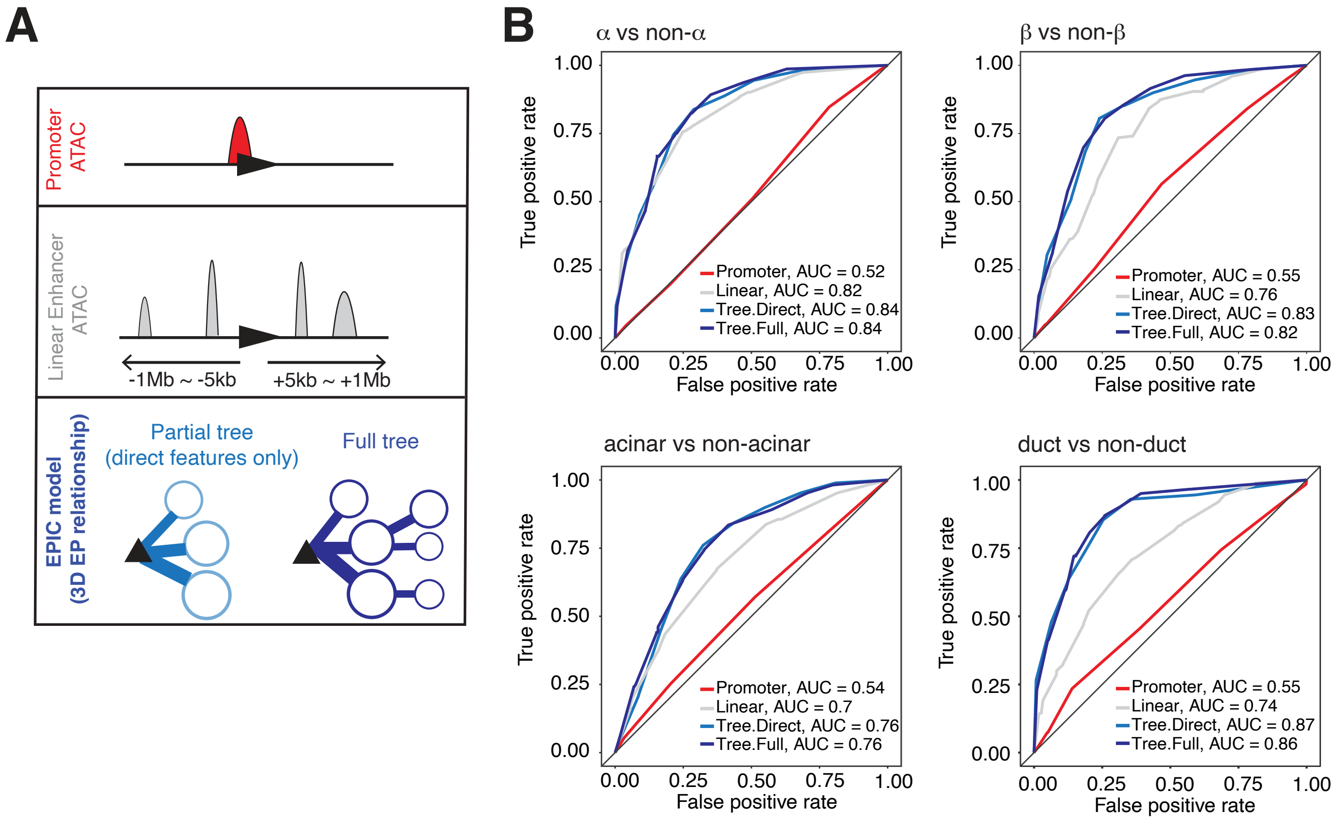

Figure S5 (related to Figure 4). Comparing EPIC's performance to alternative models.
(A) Cartoon illustrating alternative predictive models using chromatin features.
(B) ROC curves showing performance results of models shown in A. The performance of tree-based models was compared to the promoter accessibility and the linear models.

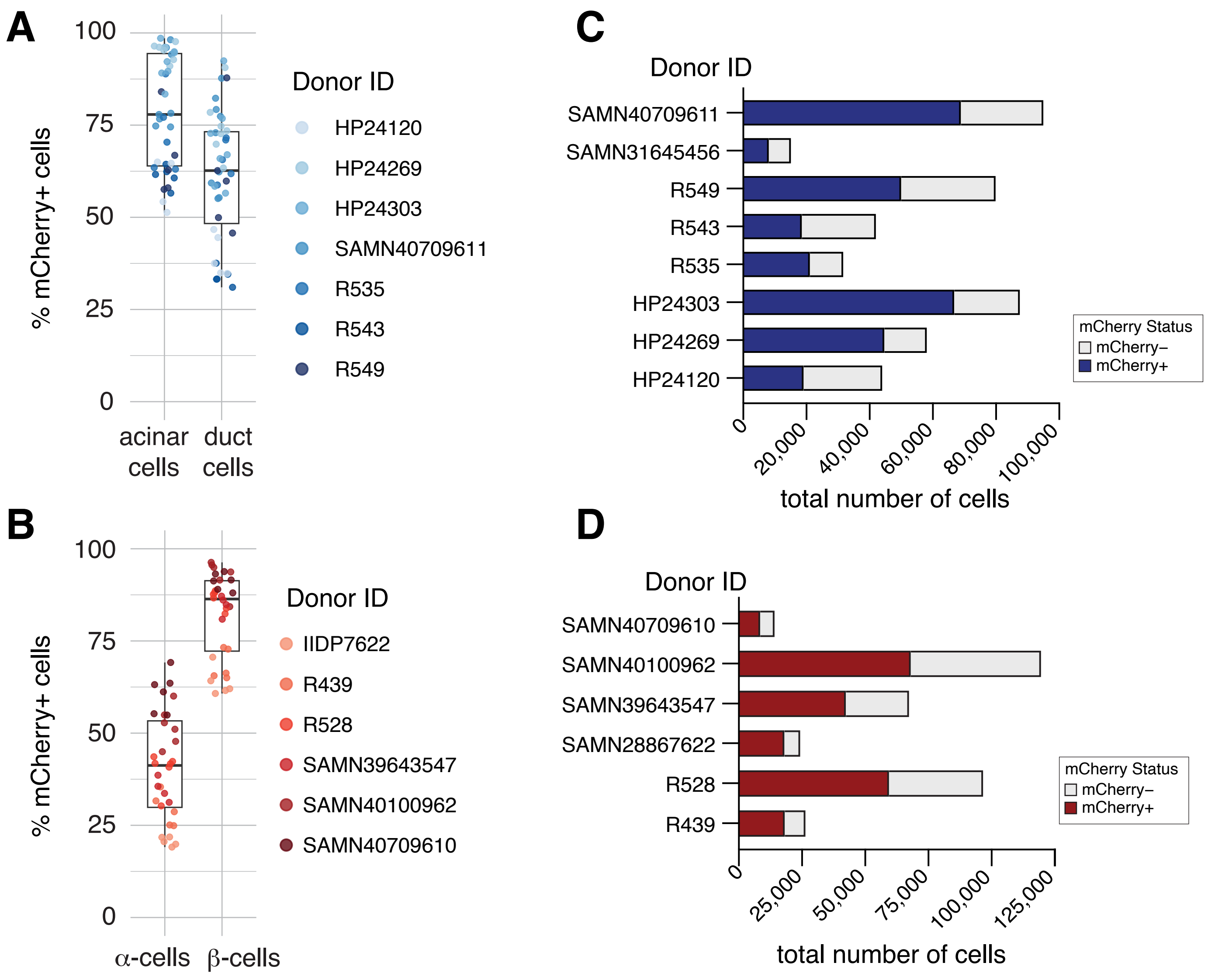

Figure S6 (related to Figure 5). Adenoviral transduction efficiency by donor.
(A-B) Box plots showing percentage of mCherry-positive cells following adenoviral transduction in exocrine (A) and endocrine (B) cells.Each point is a gRNA condition per donor, color-coded by donor. Boxes show median and interquartile range, whiskers extend to 1.5× IQR, all data points are overlaid. (C-D) Total number of mCherry-positive and mCherry-negative cells quantified per donor in exocrine (C) and endocrine (D) cells.

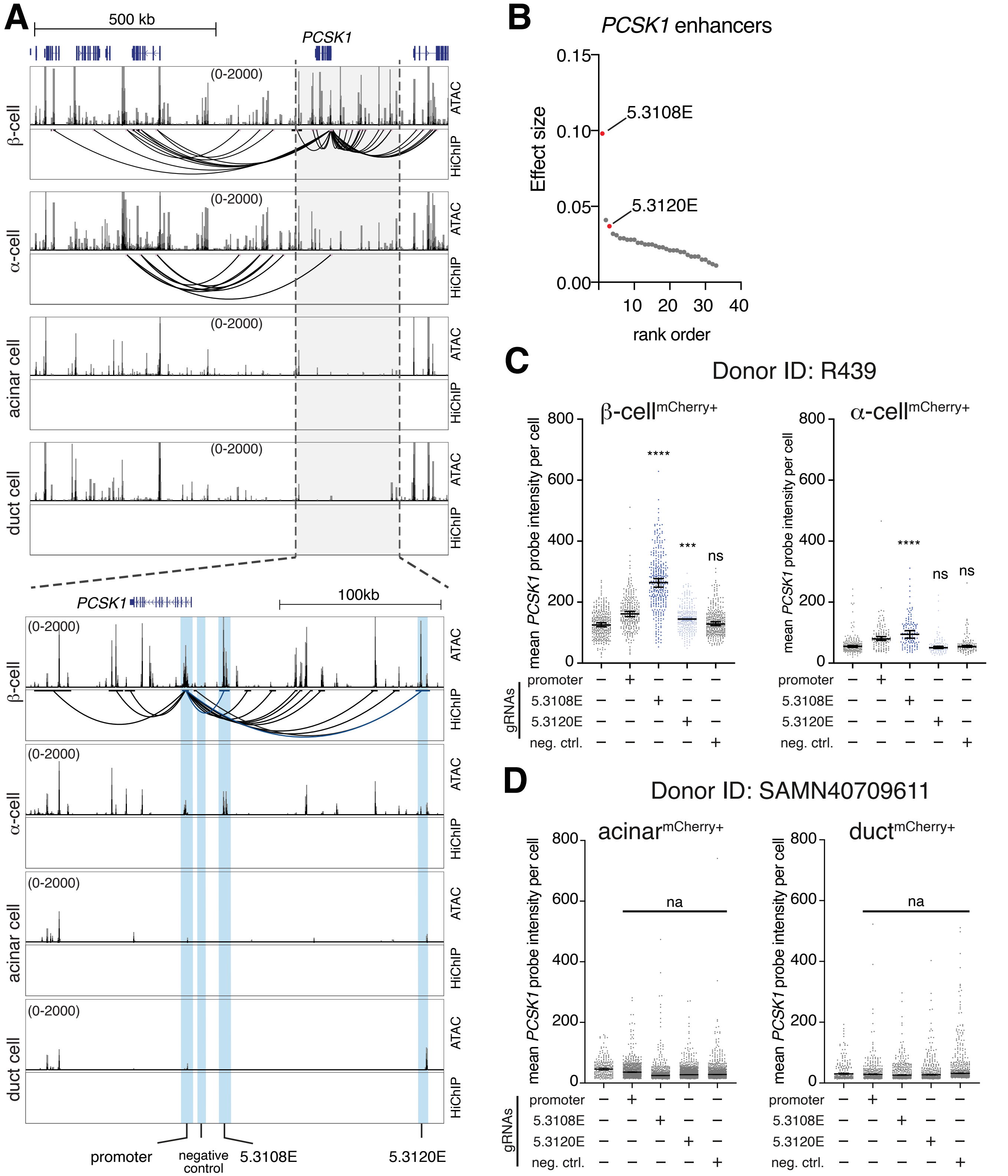

Figure S7 (related to Figure 5). EPIC enhancer ranking and chromatin profiles at the *PCSK1* locus.
(A) UCSC tracks of ATAC-seq peaks and HiChIP loops at *PCSK1* across pancreas cell types. Highlighted regions were targeted using CRISPRa. Bottom panels zoom on β-cell enhancer-promoter interactions. (B) Scatter plot of EPIC ranked effect sizes for β-cell *PCSK1* enhancers; CRISPR-tested enhancers in red. (C-D) RNA FISH quantification of *PCSK1* transcripts in α-, β-cells (C); acinar and duct cells (D). One-way ANOVA with Dunnett's test. ****, *P*-value < 0.0001; ***, *P*-value < 0.001; ns— not significant; na— no activation. Dots represent single-cell measurements grouped by gRNA condition; lines show medians, error bars show 95% confidence intervals.Complete quantification of all donors is provided in Table S9.

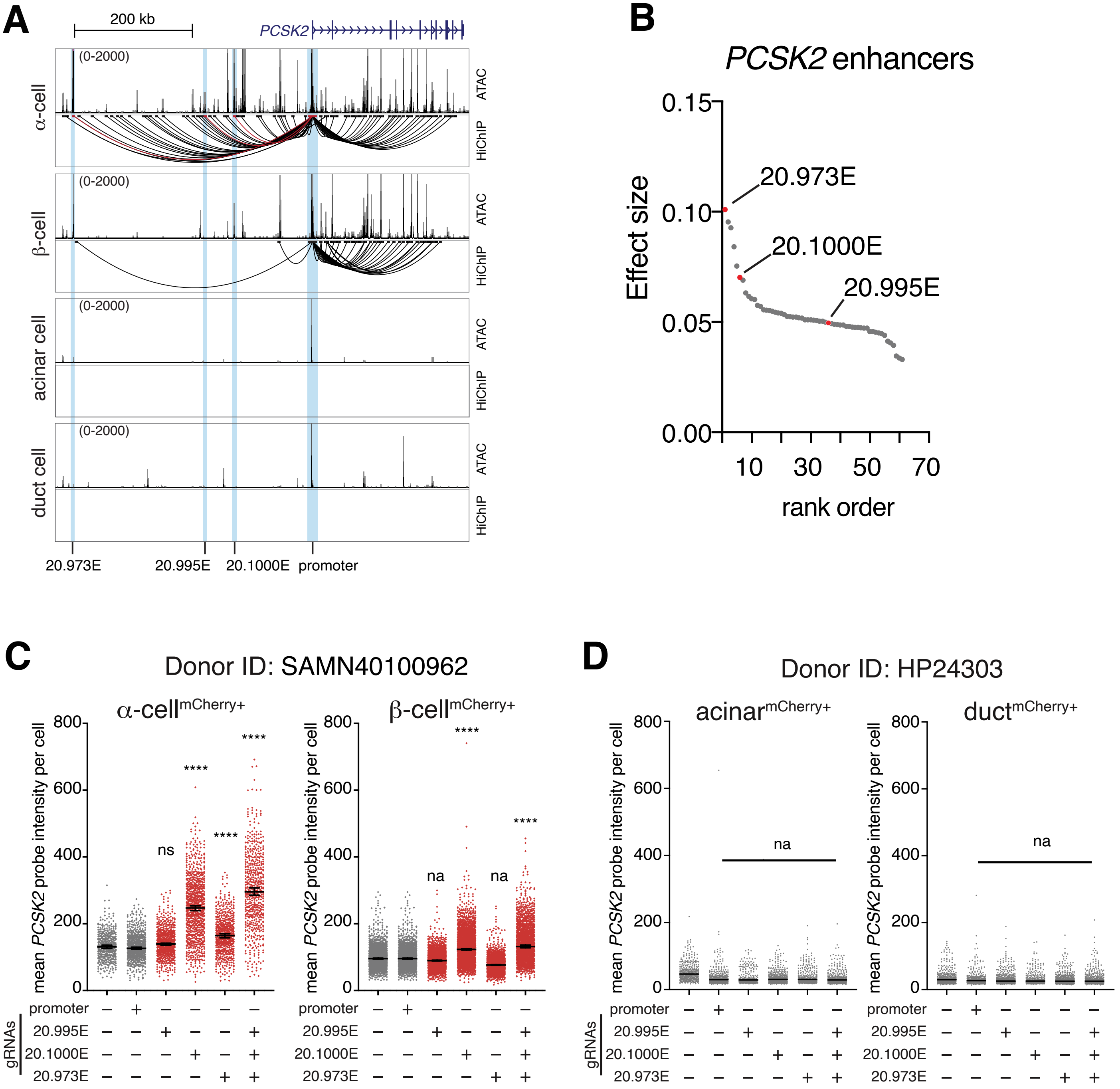

Figure S8 (related to Figure 5). EPIC enhancer ranking and chromatin profiles at the *PCSK2* locus.
(A) UCSC tracks of ATAC-seq peaks and HiChIP loops at *PCSK2* across pancreas cell types. Highlighted regions were targeted using CRISPRa. (B) Scatter plot of EPIC ranked effect sizes for α-cell *PCSK2* enhancers; CRISPR-tested enhancers in red. (C-D) RNA FISH quantification of *PCSK2* transcripts in α-, β-cells (C); acinar and duct cells (D). One-way ANOVA with Dunnett's test. ****, P-value < 0.0001; ***, P-value < 0.001; ns— not significant; na— no activation. Dots represent single-cell measurements grouped by gRNA condition; lines show medians, error bars show 95% confidence intervals.Complete quantification of all donors is provided in Table S9.

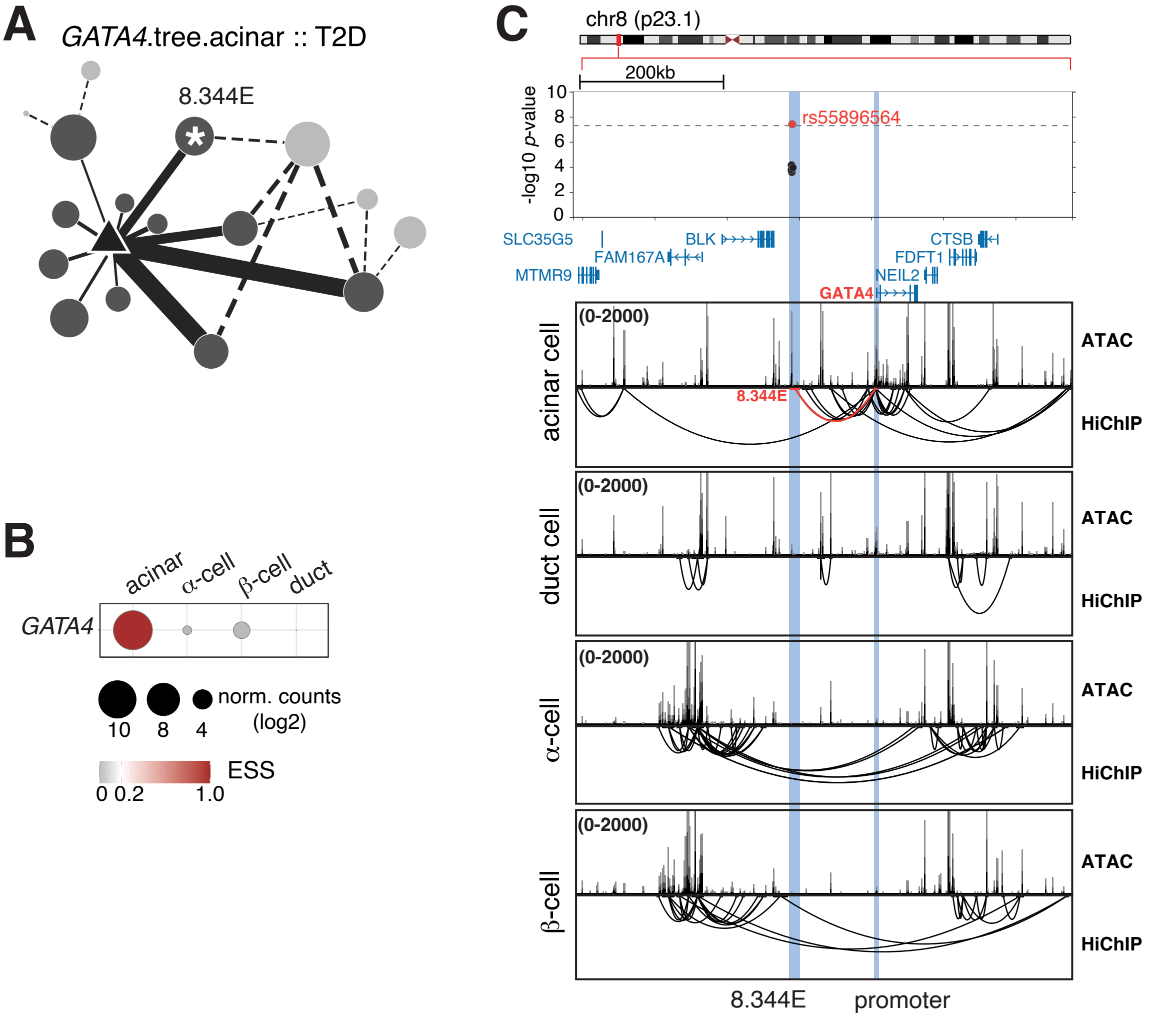

Figure S9 (related to Figure 6). Enhancer tree links type 2 diabetes risk SNP to *GATA4* in acinar cells. (A) Tree model of *GATA4* enhancer-promoter interactions in acinar cells. Node 8.344E (asterisk) overlaps T2D-associated SNP. (B) Gene expression specificity and abundance of *GATA4* across pancreas cell types. Circle size indicates transcript abundance, and color indicates expression specificity score (ESS). (C) Top: LocusZoom plot showing the association strength of SNPs at the chr8p23.1 locus for type 2 diabetes. Bottom: UCSC genome browser tracks showing ATAC-seq peaks and H3K27ac HiChIP loops across pancreas cell types. Loop connecting enhancer 8.344E to *GATA4* are present in acinar cells but absent in other cell types.

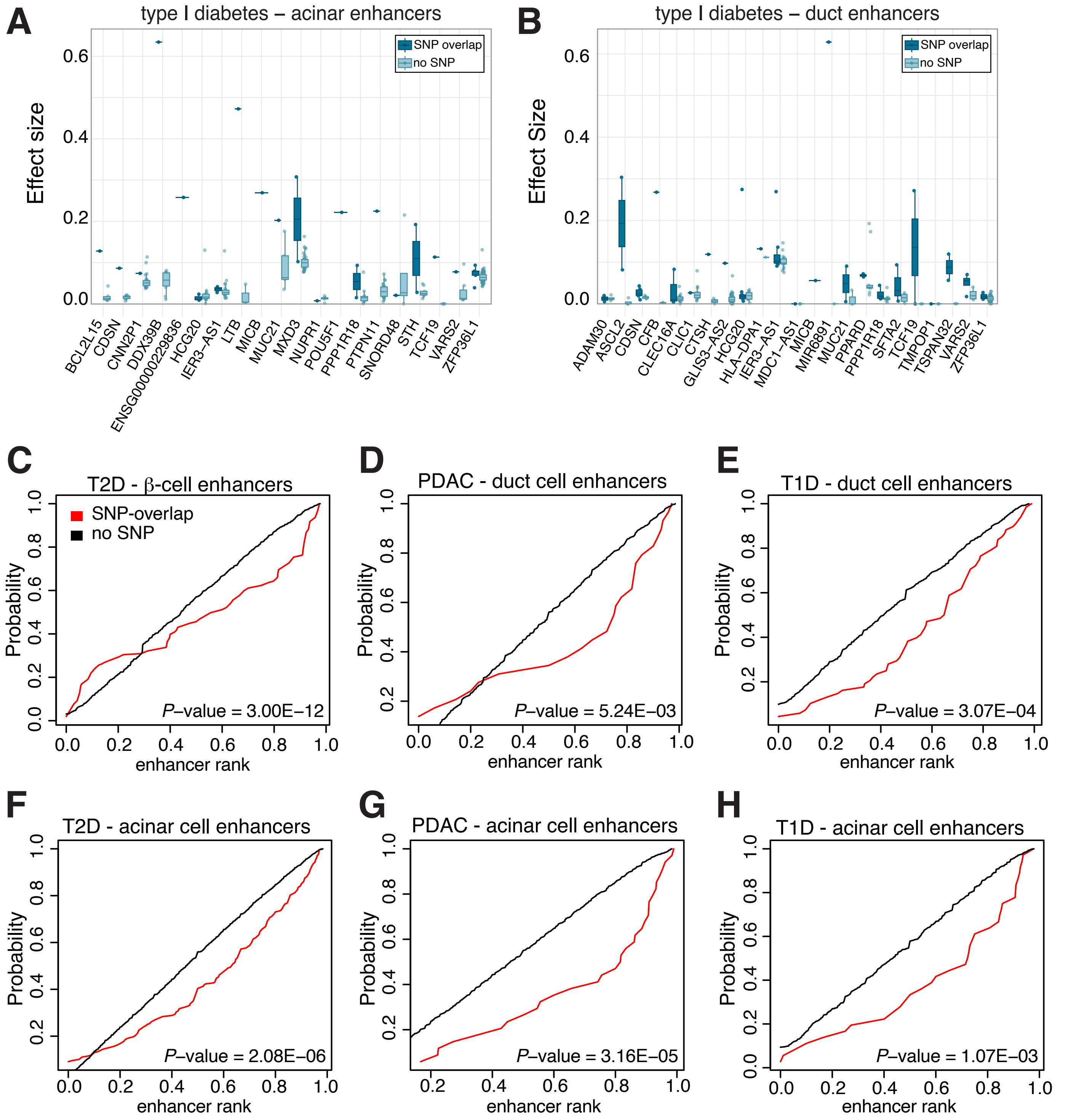

Figure S10 (related to Figure 6). Disease-associated SNPs localize to EPIC-prioritized enhancers.
(A-B) Distribution of effect sizes for enhancers in acinar (A) and (B) duct cells. Enhancers are grouped by gene, with dark blue points representing enhancers that overlap with a T1D risk SNP and light blue points indicating those without an overlapping SNP. The whiskers extend to the most extreme non-outlier points. (C–H) Cumulative distributions of EPIC ranks for SNP-overlapping (red) vs non-overlapping (black) enhancers across six trait-cell pairs: (C) T2D - β-cell, (F) T2D - acinar, (D) PDAC - duct, (G) PDAC - acinar, (E) T1D - duct, (H) T1D - acinar. *P*-values shown after Kolmogorov-Smirnov test. Normalized enhancer rank scale: 1- top-ranking, 0 - bottom-ranking.

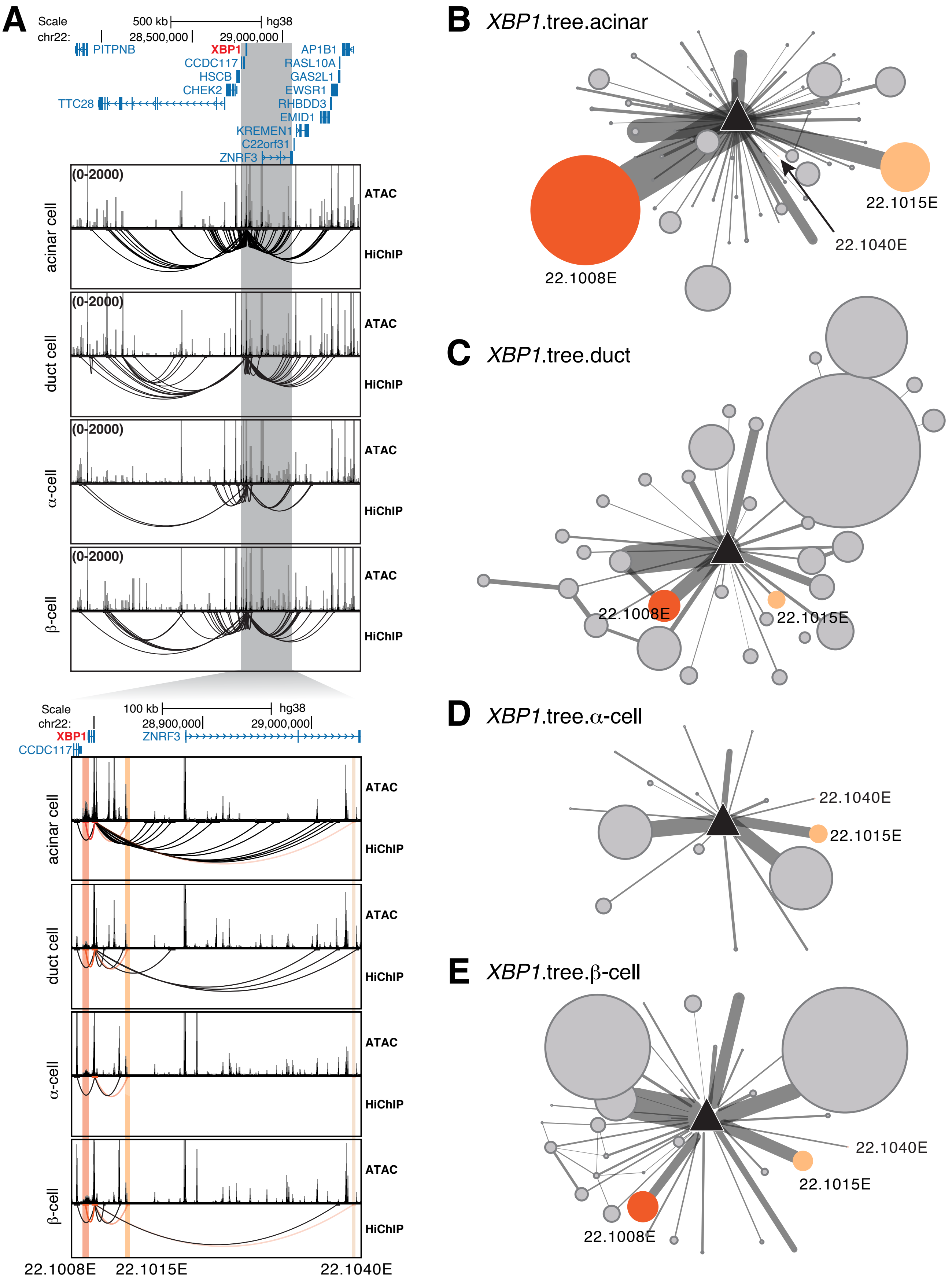

Figure S11 (related to Figure 6). Chromatin profiles and enhancer trees at *XBP1* locus across pancreas cell types. (A) UCSC tracks of ATAC-seq peaks and HiChIP loops in acinar, duct, α-, and β-cells. Shaded region: enhancer cluster overlapping PDAC SNPs. Lower panel zooms on top-, mid-, low-ranking enhancers predicted by EPIC. (B–E) Enhancer trees of *XBP1* for acinar (B), duct (C), α- (D), and β-cells (E). Node size represents ATAC-seq tag density, edge width is HiChIP interaction frequency. CRISPR-tested enhancers are highlighted and labeled.

Table S1 (related to Figure 1). Summary of donor demographics and associated assays.

Donor ID	Age	Sex	Body Mass Index (BMI)	Assays
GW19244	58	female	15.1	ATAC-seq
GW20010	64	male	29.9	ATAC-seq
R290	74	female	35.8	ATAC-seq, HiChIP
R299	44	female	25.4	ATAC-seq, HiChIP
R300	30	female	25.3	HiChIP
R303	56	female	24.1	HiChIP
R325	50	male	30.3	HiChIP
R341	42	male	30	HiChIP
R356	45	female	29.7	ATAC-seq
R357	64	male	24.3	ATAC-seq
R360	51	female	26.1	ATAC-seq
R361	65	female	20.8	ATAC-seq
R362	54	male	29.4	ATAC-seq
R364	58	male	27.8	ATAC-seq
SAMN10391370	48	male	24.4	ATAC-seq, HiChIP
SAMN10737781	66	male	27.2	HiChIP
SAMN10913630	53	male	25.1	HiChIP
SAMN10977276	52	male	27.2	HiChIP
SAMN11773852	30	male	27.8	HiChIP
SAMN11788169	44	female	29.5	HiChIP
SAMN12572087	21	male	21.2	HiChIP
SAMN12736601	46	male	45.2	ATAC-seq
SAMN13047843	57	male	21.3	ATAC-seq
SAMN13086797	42	male	25	ATAC-seq
SAMN13283508	58	male	26.6	ATAC-seq, HiChIP
SAMN13898077	49	male	26.1	ATAC-seq
SAMN14132340, SAMN14124567	31	male	27	ATAC-seq
SAMN14327036	48	male	29.2	ATAC-seq
SAMN28867622	36	male	29.6	CRISPR/HCR
R439	57	female	26	CRISPR/HCR
SAMN31645456	68	male	30.7	CRISPR/HCR
R528	53	male	34.5	CRISPR/HCR
SAMN40709610, SAMN40709611	55	female	28.6	CRISPR/HCR
R535	27	male	24.8	CRISPR/HCR
R543	54	female	20.2	CRISPR/HCR
R549	54	male	26.1	CRISPR/HCR

Donor ID	Age	Sex	Body Mass Index (BMI)	Assays
SAMN40100962	58	male	28.8	CRISPR/HCR
SAMN39643547	49	female	21.2	CRISPR/HCR
HP24120	66	female	28.6	CRISPR/HCR
HP24269	43	male	27.1	CRISPR/HCR
HP24303	49	male	24.5	CRISPR/HCR

Table S11 (related to Figures 5 and 6). gRNA sequences and CARGO arrays used in this study.

gRNA Sequence	Target Region	Target gene
GAGCTCCCTAGAGAGTCGCG	promoter	*PCSK1*
GATCCGAATGGTATTCCCGG	promoter	*PCSK1*
GCATAGATCGCGCCCTCGGG	promoter	*PCSK1*
GTTTGTACAGAAGATGAGGG	5.3108E	*PCSK1*
GTAAAGTCCCTGTCAAAATG	5.3108E	*PCSK1*
GTGGGATGATCCTCTTCAAG	5.3108E	*PCSK1*
GCTTCATTGTACTTCCATAG	5.3108E	*PCSK1*
GACCCAGGCATGTTTTACCT	5.3108E	*PCSK1*
GCACTGATCTACTTCAAGCT	5.3108E	*PCSK1*
GTTGGCAGTCAAATTCCTAT	5.3108E	*PCSK1*
GTTTATTTAAGTCAGTAACT	5.3108E	*PCSK1*
GGTGATTTATGGCTCTACCT	5.3108E	*PCSK1*
GTCCAATGAGGAGGGCCAGA	5.3120E	*PCSK1*
GTGATAAGTGGCAGATAGGA	5.3120E	*PCSK1*
GTGGCAGGTGACTATACTTT	5.3120E	*PCSK1*
GGTGAGTCAGAGCAAACTAA	5.3120E	*PCSK1*
GCTGCCCATACATAACAAAA	5.3120E	*PCSK1*
GAGAGGCCAGCAGTACTAGG	5.3120E	*PCSK1*
GTAGGGCCATACATACACAT	negative control	*PCSK1*
GCTAAGACCAGCAAGCCCAG	negative control	*PCSK1*
GAAGTTCACCCTGTGAGCCG	negative control	*PCSK1*
GCGGGCAGGGGTGCGAATGTG	promoter	*PCSK2*
GAGCTGTAATCAGGCCAGCGG	promoter	*PCSK2*
GGAGTCCGCCAGCGGCCAATG	promoter	*PCSK2*
GCGAGAATCCAAGCACCCAG	20.973E	*PCSK2*
GGTGCTGTAGGGTATTGAGC	20.973E	*PCSK2*
GTAATTATTTTGGCACAGGT	20.973E	*PCSK2*
GATCTATTCCTACAGTCCCA	20.995E	*PCSK2*
GTGCATTCAACTCAAGAGAG	20.995E	*PCSK2*
GCCTTGTTTTGAAGGGTAGA	20.995E	*PCSK2*
GCTCCTAAAGGAAAACCAGT	20.1000E	*PCSK2*
GATCTTTTCAGCCTGCAGAC	20.1000E	*PCSK2*
GTAACAGTTGTTCCTTCAGT	20.1000E	*PCSK2*
GCAGAACTTTAGGGGTCCCGT	promoter	*XBP1*
GTCCCTGGCCAAAGGTACTTG	promoter	*XBP1*
GCACGCGACGCTGGCCAATCG	promoter	*XBP1*
GCTACACCTGGCCTACACCAA	22.1008E	*XBP1*
GTGGTAAGCTGGGTAACCCAG	22.1008E	*XBP1*

**gRNA Sequence**	**Target Region**	**Target gene**
GCCTCCAGTCACTACATGAA	22.1008E	*XBP1*
GAGCGTTAGGCTCACTTCAGT	22.1008E	*XBP1*
GCACTGTTTGCCAAATCAGAC	22.1015E	*XBP1*
GTGACTGGACTTTGTTTCTAG	22.1015E	*XBP1*
GGTGCCATCGCCAACCGCAT	22.1015E	*XBP1*
GGATGTGAAGATACCCTTGT	22.1040E	*XBP1*
GTTTTCCCGCAATAAAGGGT	22.1040E	*XBP1*
GTGAGACCTCCACGTTCCGGG	22.1040E	*XBP1*
**CARGO array names**	**Target Region**	**Target gene**
PCSK1_Pr	promoter	*PCSK1*
PCSK1_B123L	5.3108E	*PCSK1*
PCSK1_B7HIR	5.3120E	*PCSK1*
PCSK1_RR1	Negative control	*PCSK1*
PCSK2_Pr	Promoter	*PCSK2*
PCSK2_A2	20.995E	*PCSK2*
PCSK2_A4	20.1000E	*PCSK2*
PCSK2_A10	20.973E	*PCSK2*
XBP1_Pr	promoter	*XBP1*
XBP1_22.1008E	22.1008E	*XBP1*
XBP1_22.1015E	22.1015E	*XBP1*
XBP1_22.1040E	22.1015E	*XBP1*

Table S12 (related to Figures 5 and 6). HCR probes and amplifiers used in this study.

Purpose	Probe sets with barcode	Amplifiers with barcode and fluorophore	Gene identifier (Accession #)
Gene target	PCSK1-B2	B2 Alexa 647	NM_000439.5
	PCSK2-B3	B3 Alexa 647	NM_002594.5
	XBP1-B2	B2 Alexa 647	NM_005080.4
Cell markers	INS-B1	B1 Alexa 488	NM 000207.3
	GCG-B1	B1 Alexa 488	NM_002054.5
	CPA1-B1	B1 Alexa 488	NM_001868.4
	SPP1-B1	B1 Alexa 488	NM_001040058.2

The Pancreatic Cancer Cohort Consortium (PanScan):

Jun Zhong[1], Demetrius Albanes[2], Gabriella Andreotti[3], Alan A Arslan[4], Laura Beane-Freeman[3], Sonja I Berndt[3], Julie E Buring[5,6], Daniele Campa[7], Federico Canzian[8], Stephen J Chanock[9], Yu Chen[10], Sandra M Colorado-Yohar[11,12,13], A. Heather Eliassen[14], J. Michael Gaziano[15,16,17], Graham G Giles[18,19,20], Phyllis J Goodman[21], Christopher A Haiman[22], Mattias Johansson[23], Verena Katzke[24], Charles Kooperberg[25], Peter Kraft[26], Manolis Kogevinas[27,28,29,30], I-Min Lee[5,6], Loic LeMarchand[31], Núria Malats[32,33], Satu Männistö[34], Marjorie L McCullough[35], Roger Milne[18,19,20], Stephen C Moore[2], Lorelei Mucci[36], Salvatore Panico[37], Alpa V Patel[35], Ulrike Peters[38], Miquel Porta[29], Francisco X Real[39,33,30], Howard D Sesso[15,6], Xiao-Ou Shu[40], Meir J Stampfer[14,41], Geoffrey S Tobias[9], Kala Visvanathan[42,43], Elisabete Weiderpass[44], Nicolas Wentzensen[9], Emily White[45,46], Chen Yuan[47], Wei Zheng[40], Jean Wactawski-Wende[48], Rachael Z Stolzenberg-Solomon[2], Brian M Wolpin[49], Laufey T Amundadottir[1]

[1]Laboratory of Translational Genomics, Division of Cancer Epidemiology and Genetics, National Cancer Institute, National Institutes of Health, Bethesda, MD, USA, [2]Metabolic Epidemiology Branch, Division of Cancer Epidemiology and Genetics, National Cancer Institute, National Institutes of Health, Bethesda, MD, USA, [3]Occupational and Environmental Epidemiology Branch, Division of Cancer Epidemiology and Genetics, National Cancer Institute, National Institutes of Health, Bethesda, MD, USA, [4]Departments of Obstetrics and Gynecology and Population Health, NYU Grossman School of Medicine, NYU Perlmutter Comprehensive Cancer Center, New York, NY, USA, [5]Division of Preventive Medicine, Department of Medicine, Brigham and Women's Hospital, Boston, MA, USA, [6]Department of Epidemiology, Harvard T.H. Chan School of Public Health, Boston, MA, USA, [7]Unit of Genetics., Department of Biology, University of Pisa, Pisa, Italy, [8]Genomic Epidemiology Group, German Cancer Research Center (DKFZ), Heidelberg, Germany, [9]Division of Cancer Epidemiology and Genetics, National Cancer Institute, National Institutes of Health, Bethesda, MD, USA, [10]Department of Population Health, NYU Grossman School of Medicine, NYU Perlmutter Comprehensive Cancer Center, New York, NY, USA, [11]Department of Epidemiology, Murcia Regional Health Council, IMIB-Arrixaca, Murcia, Spain, [12]CIBER Epidemiología y Salud Pública (CIBERESP), Spain, [13]Research Group on Demography and Health, National Faculty of Public Health,, University of Antioquia, Medellín, Colombia, [14]Department of Epidemiology, Harvard T. H. Chan School of Public Health, Boston, MA, USA, [15]Division of Preventive Medicine, Brigham and Women's Hospital, Boston, MA, USA, [16]Division of Aging, Brigham and Women's Hospital, Boston, MA, USA, [17]Boston VA Healthcare System, Boston, MA, USA, [18]Cancer Epidemiology Division, Cancer Council Victoria, Melbourne, VIC, Australia, [19]Centre for Epidemiology and Biostatistics, Melbourne School of Population and Global Health, The University of Melbourne, Parkville, VIC, Australia, [20]Precision Medicine, School of Clinical Sciences at Monash Health, Monash University, Melbourne, VIC, Australia, [21]SWOG Statistical Center, Fred Hutchinson Cancer Research Center, Seattle, WA, USA, [22]Department of Preventive Medicine, Keck School of Medicine, University of Southern California, Los Angeles, CA, [23]Genomic Epidemiology Branch, International Agency for Research on Cancer (IARC/WHO), Lyon, France, [24]Division of Cancer Epidemiology, German Cancer Research Center (DKFZ), Heidelberg, Germany, [25]Division of Public Health Sciences, Fred Hutchinson Cancer Research Center, Seattle, WA, [26]Trans-Divisional Research Program (TDRP), Division of Cancer Epidemiology and Genetics, National Cancer Institute, National Institutes of Health, Bethesda, MD, USA, [27]ISGlobal, Centre for Research in Environmental Epidemiology (CREAL), Barcelona, Spain, [28]CIBER Epidemiología y Salud Pública (CIBERESP), Barcelona, Spain, [29]Hospital del Mar Institute of Medical Research (IMIM), Universitat Autònoma de Barcelona, Barcelona, Spain, [30]Universitat Pompeu Fabra (UPF), Barcelona, Spain, [31]Cancer Epidemiology Program, University of Hawaii Cancer Center, Honolulu, HI, USA, [32]Genetic and Molecular Epidemiology Group, Spanish National Cancer Research Center (CNIO), Madrid, Spain, [33]CIBERONC, Madrid, Spain, [34]Department of

Public Health and Welfare, Finnish Institute for Health and Welfare (THL), Helsinki, Finland, [35]Department of Population Science, American Cancer Society, Atlanta, GA, USA, [36]Department of Epidemiology, Harvard T. H. Chan School of Public Health, Boston, MA, [37]Dipartimento Di Medicina Clinica E Chirurgia, Federico II University, Naples, Italy, [38]Division of Public Health Sciences, Fred Hutchinson Cancer Center, Seattle, WA, USA, [39]Epithelial Carcinogenesis Group, Molecular Oncology Programme, Spanish National Cancer Research Center (CNIO), Madrid, Spain, [40]Division of Epidemiology, Department of Medicine, Vanderbilt Epidemiology Center, Vanderbilt-Ingram Cancer Center, Vanderbilt University School of Medicine, Nashville, TN, USA, [41]Department of Nutrition, Harvard T. H. Chan School of Public Health, Boston, MA, USA, [42]Department of Epidemiology, Johns Hopkins School of Public Health, Baltimore, MD, [43]Department of Oncology, Sidney Kimmel Comprehensive Cancer Center, Johns Hopkins School of Medicine, Baltimore, MD, [44]International Agency for Research on Cancer (IARC/WHO), Lyon, France, [45]Division of Public Health Sciences, Fred Hutchinson Cancer Research Center, Seattle, WA, USA, [46]Department of Epidemiology, University of Washington, Seattle, WA, USA, [47]Department of Medical Oncology, Dana-Farber Cancer Institute, Harvard Medical School, Harvard University, Boston, MA, [48]Department of Epidemiology and Environmental Health, University of Buffalo, Buffalo, NY, USA, [49]Department of Medical Oncology, Dana-Farber Cancer Institute, Harvard Medical School, Harvard University, Boston, MA, USA

Pancreatic Cancer Case-Control Consortium (PanC4):

Samuel O Antwi[1], Paige M Bracci[2], Steven Gallinger[3], Michael Goggins[4], Manal Hassan[5], Elizabeth A Holly[2], Rayjean J Hung[3], Donghui Li[5], Núria Malats[6,7], Rachel E Neale[8], Kari G Rabe[9], Harvey A Risch[10], Herbert Yu[11], Alison P Klein[12,13,4]

[1]Department of Quantitative Health Sciences, Mayo Clinic College of Medicine, Jacksonville, FL, USA, [2]Department of Epidemiology and Biostatistics, University of California, San Francisco, San Francisco, CA, USA, [3]Lunenfeld-Tanenbaum Research Institute, Sinai Health System and University of Toronto, Toronto, Canada, [4]Department of Pathology, Sol Goldman Pancreatic Cancer Research Center, Johns Hopkins School of Medicine, Baltimore, MD, USA, [5]Department of Gastrointestinal Medical Oncology, University of Texas MD Anderson Cancer Center, Houston, TX, USA, [6]Genetic and Molecular Epidemiology Group, Spanish National Cancer Research Center (CNIO), Madrid, Spain, [7]CIBERONC, Madrid, Spain, [8]Population Health Program, QIMR Berghofer Medical Research Institute, Brisbane, Australia, [9]Department of Quantitative Health Sciences, Mayo Clinic College of Medicine, Rochester, MN, USA, [10]Department of Chronic Disease Epidemiology, Yale School of Public Health, New Haven, CT, USA, [11]Epidemiology Program, University of Hawaii Cancer Center, Honolulu, HI, USA, [12]Department of Epidemiology, Johns Hopkins School of Public Health, Baltimore, MD, USA, [13]Department of Oncology, Sidney Kimmel Comprehensive Cancer Center, Johns Hopkins School of Medicine, Baltimore, MD, USA
